# ^18^F-Flortaucipir in TDP-43 associated frontotemporal dementia

**DOI:** 10.1038/s41598-019-42625-9

**Published:** 2019-04-15

**Authors:** R. Smith, A. F. Santillo, M. Landqvist Waldö, O. Strandberg, D. Berron, S. Vestberg, D. van Westen, J. van Swieten, M. Honer, O. Hansson

**Affiliations:** 1 Clinical Memory Research Unit, Department of Clinical Sciences, Malmö, Lund University, Lund, Sweden; 2 Department of Neurology, Skåne University Hospital, Lund, Sweden; 3 Clinical Sciences Helsingborg, Department of Clinical Sciences, Lund, Lund University, Lund, Sweden; 4 Department of Psychology, Lund University, Lund, Sweden; 5 Department of Neurology, Erasmus Medical Centre, Rotterdam, The Netherlands; 6 Roche Pharmaceutical Research and Early Development, Neuroscience Translational Technologies, Roche Innovation Center, Basel, Switzerland; 7 Memory Clinic, Skåne University Hospital, Malmö, Sweden

## Abstract

Retention of ^18^F-Flortaucipir is reportedly increased in the semantic variant of primary progressive aphasia (svPPA), which is dominated by TDP-43 pathology. However, it is unclear if ^18^F-Flortaucipir is also increased in other TDP-43 diseases, such as bvFTD caused by a *C9orf72* gene mutation. We therefore recruited six *C9orf72* expansion carriers, six svPPA patients, and 54 healthy controls. All underwent ^18^F-Flortaucipir PET and MRI scanning. Data from 39 Alzheimer’s Disease patients were used for comparison. PET tracer retention was assessed both at the region-of-interest (ROI) and at the voxel-level. Further, autoradiography using ^3^H-Flortaucipir was performed. SvPPA patients exhibited higher ^18^F-Flortaucipir retention in the lateral temporal cortex bilaterally according to ROI- and voxel-based analyses. In *C9orf72* patients, ^18^F-Flortaucipir binding was slightly increased in the inferior frontal lobes in the ROI based analysis, but these results were not replicated in the voxel-based analysis. Autoradiography did not show specific binding in svPPA cases or in *C9orf72*-mutation carriers. In conclusion, temporal lobe ^18^F-Flortaucipir retention was observed in some cases of svPPA, but the uptake was of a lower magnitude compared to AD dementia. *C9orf72*-mutation carriers exhibited none or limited ^18^F-Flortaucipir retention, indicating that ^18^F-Flortaucipir binding in TDP-43 proteinopathies is not a general TDP-43 related phenomenon.

## Introduction

*Ante mortem* identification of the underlying pathology in frontotemporal dementias (FTD) is challenging. Semantic variant primary progressive aphasia (svPPA), that is part of the frontotemporal disease spectrum, is clinically associated with anomia and difficulties in single word comprehension, often accompanied by visual associative agnosia^1–3^. Histologically, specimens from people with svPPA most often exhibit TDP-43 pathology, however tauopathies such as Alzheimer’s Disease (AD) and Pick’s disease may occasionally cause svPPA^1,3,4^. Behavioral variant FTD (bvFTD) due to hexanucleotide expansions in the *C9orf72*-gene is strongly associated to TDP-43 pathology^4,5^.

Several positron emission tomography (PET) radioligands for the microtubule associated protein tau have been developed in recent years^6^. These include the family of THK compounds^7^, ^11^C-PBB3^8^ as well as the most commonly used ^18^F-Flortaucipir^9^. This tracer primarily detects the mixed 3R/4R tau pathology related to AD^10–18^. *In vivo*, ^18^F-Flortaucipir retention has unexpectedly shown an increased retention in svPPA^19,20^, and recently a report indicated temporal retention of Flortaucipir in a *C9orf72*-mutation carrier^21^. By contrast, *in vitro*, using autoradiography, ^18^F-Flortaucipir did not bind to TAR DNA-binding protein 43 (TDP-43) pathology^13^ or only showed minimal binding^12,15^ in some cases. The TDP-43 pathology is subclassified into different categories depending on the distribution of TDP-43 aggregates in the cell bodies and in the neurites; type A, where neuritic pathology roughly equals intrasomal pathology; type B, where neuritic pathology is less frequent than intrasomal pathology; and type C, where neuritic pathology is more common than intrasomal pathology^4,5^. In svPPA the pathology is mainly type C^4,5^. So far only TDP-43-cases with type A and type C pathology have been studied using ^18^F-Flortaucipir autoradiography^12,13,15^. It is at this stage not fully clear whether the retention of radiotracer detected in svPPA represents binding to true tau-aggregates, whether it binds to TDP-43 or whether this is a retention due to non-tau, non-TDP-43 related neurodegeneration^19,20^. To further study whether ^18^F-Flortaucipir binds to TDP-43 related pathology we recruited a group of svPPA patients along with a group of patients with bvFTD due to hexanucleotide expansions in the *C9orf72*-gene, known to be strongly related to TDP-43 pathology of type A and B^4,5^. Since the genetic background is known in patients with *C9orf72*-mutations, we can assume that the mutation carriers have a more predictable TDP-43 pathology underlying their symptoms, allowing us to study the effect of TDP-43 pathology on ^18^F-Flortaucipir retention.

## Material and Methods

### Participants

**Figure 1:**
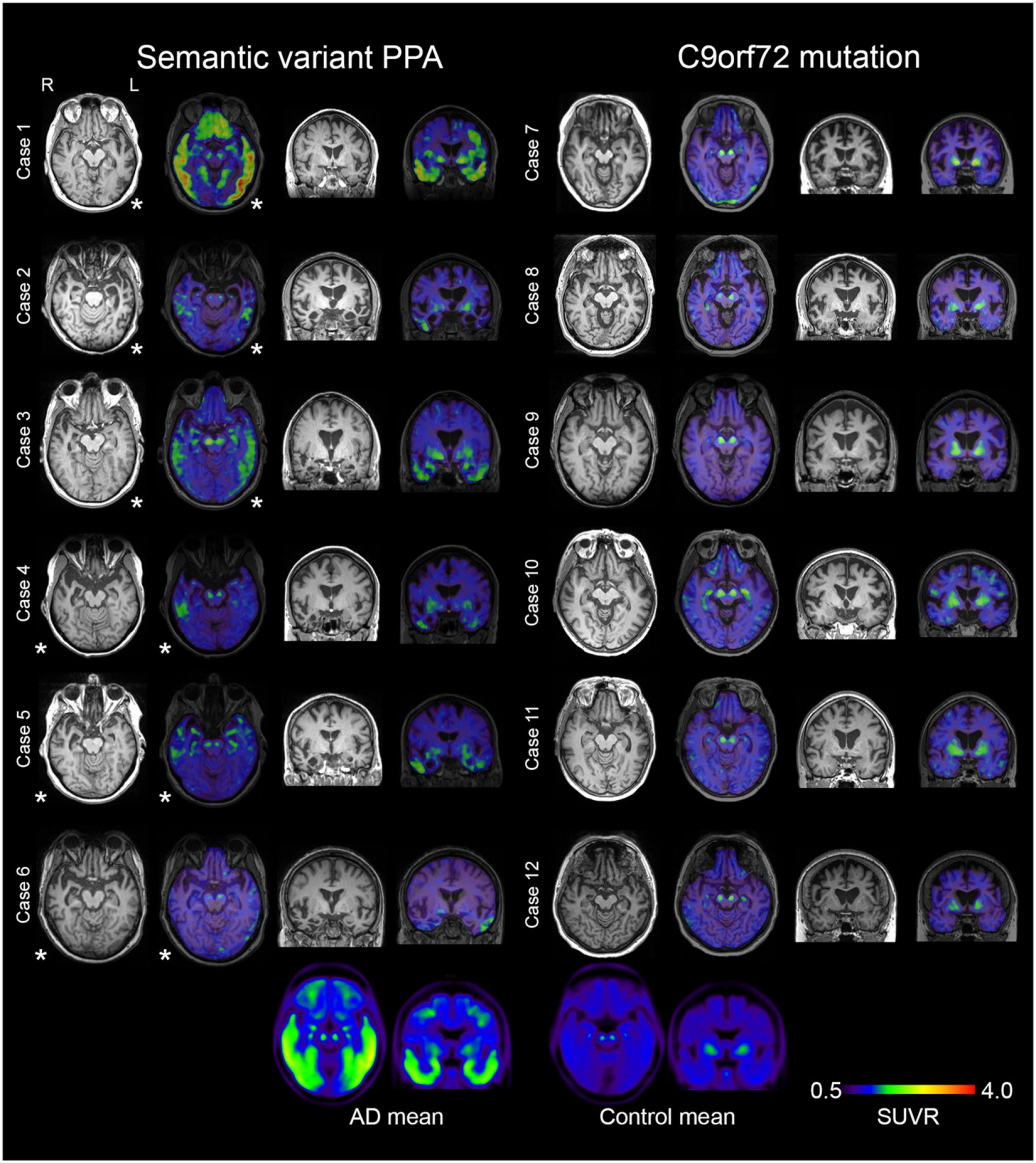
MRI and ^18^F-Flortaucipir PET scans in svPPA and *C9orf72*-mutation carriers. Transversal and coronal slices of structural (T1-mprage) MRI scans and ^18^F-Flortaucipir PET scans are shown for all included subjects with semantic variant primary progressive aphasia (svPPA) and bvFTD due to a hexanucleotide expansion in the *C9orf72*-gene. Case numbers correspond to case numbers in Table 1. Below the individual images are mean SUVR-images of 40 AD patients and the 54 controls in MNI-space, included for comparative purposes. The images are presented in radiological orientation (left in the image corresponds to patient right). Asterisks indicate the most affected side in the svPPA cases. Scale bar for PET-scans represents standardized uptake value ratios (SUVR). An identical figure with SUVR range 0.5–3 is included as Supplementary Fig. 5.

The study participants were recruited from the Neurology and Memory clinics at Skåne University Hospital and from the memory clinic at Ängelholm Hospital, Sweden as part of the ongoing Swedish BioFINDER study (www.biofinder.se). Controls within the BioFINDER-study were enrolled from the prospective Malmö Diet and Cancer study cohort^22^. We included: 1) six patients with svPPA diagnosed according to international criteria^2^, three with left sided dominant syndrome (L-svPPA) and three with the right sided dominant syndrome (“right semantic dementia”, R-SD)^19,23^. All patients had undergone a neuropsychological examination by a neuropsychologist, and all diagnoses were imaging supported (i.e. all patients had an atrophy pattern on MRI consistent with the diagnosis^24^; results of CSF and amyloid imaging (see below) were not used for diagnosis); 2) six symptomatic patients with bvFTD fulfilling criteria for bvFTD with definite FTLD pathology^25^, all carrying *C9orf72* hexanucleotide mutations; and, 3) 54 neurologically healthy, age-matched controls (Table 1). All patients were assessed by a physician, experienced in neurocognitive disorders. Aβ-status was determined using either Aβ_42_/pTau values in cerebrospinal fluid (CSF) or using ^18^F-Flutemetamol PET. CSF was collected and handled as previously described^26^. Amyloid positivity was defined as ^18^F-Flutemetamol Berkley neocortical composite score^27^ >0.693, with a combined cerebellar, brain stem and white matter reference^28^, or as having an Aβ_42_/pTau ratio < 8.0 (determined by Mixture modelling)^29^. For visual comparison, mean SUVR images of 39 AD patients participating in the BioFINDER study were included in Fig. 1, and SUVR-values in the regions studied in Supplementary Fig. 4. All AD patients fulfilled the McKhann-criteria for AD^30^.

**Table 1:** Demographical, clinical and cognitive data.

Case	Diagnosis	Demographics (Age, gender, handedness, disease duration, education (years))	Presenting features	MMSE	Amyloid status (^18^F-Flutemetamol Berkeley score, cut off >0.693)	CSF-Aβ_42_/ pTau cut off <8.0
1	L-svPPA	76, M, Right, 7, 22	Anomia, impaired single word comprehension, semantic paraphasia	20	**0**.**738**	**1**.**3**
2	L-svPPA	66, M, Right, 8, 13	Anomia, impaired single word comprehension, sematic paraphasia, logorrhea	10	0.642	29.2
3	L-svPPA	64, M, Right, 7, 9	Anomia, impaired single word comprehension, prosopagnosia	29	**0**.**789**	**5**.**5**
4	R-SD	60, F, Right, 8, 21	Bipolar II disorder, dysexecutivity, memory loss, mental rigidity and repetitive behaviours, proposagnosia, visual agnosia and anomia	25	0.637	23.2
5	R-SD	73, F, Right, 7, 16	Social inappropriateness, mental rigidity and repetitive behaviours, prosopagnosia, reduced empathy, anomia and impaired single word comprehension	30	**0**.**869**	**4**.**2**
6	R-SD	60, F, Right, 7, 11	Distractability, social inappropriateness, mental rigidity and repetitive behaviours, prosopagnosia, anomia and impaired single word comprehension	24	0.558	18.8
7	FTD-*C9orf72*	69, F, Right, 2 14	Apathy, dysexecutivity, loss of insight, visual hallucinations	24	**0**.**706**	10.1
8	FTD-*C9orf72*	68, M, Right, 2, 10	Apathy, dysexecutivity, hyperorality, flattening of affect, loss of insight	N/A	N/A	N/A
9	FTD-*C9orf72*	66, F, Right, 5, N/A	Non-fluent aphasia, reduced empathy, apathy, hyperorality, and paranoid delusions	21	N/A	13.9
10	FTD-*C9orf72*	66, F, Right, 3, N/A	Apathy, affective flattening, non-fluent aphasia, motor stereotypies	20	N/A	25.1
11	FTD-*C9orf72*	61, F, Right, 3, 12	Mild executive deficits, irritability, apathy and visual hallucinations	24	N/A	N/A
12	FTD-*C9orf72*	66, F, Right, 4, 9	Apathy, affective flatting, altered eating habits, loss of insight, paranoid delusions	21	N/A	16.4
	Controls	75 ± 5 yr; 26 F/28 M; N/A; 12 ± 4 yr	—	29 ± 1	26 abnormal 27 normal	21 abnormal 33 normal

F – female; FTD – frontotemporal dementia; L – left; M – male; N/A – not available; R – right; SD – semantic dementia; svPPA – semantic variant primary progressive aphasia. Control means are shown ± 1 standard deviation.

Informed written consent was obtained from all patients prior to inclusion in the study. All procedures were approved by the Regional ethics committee at Lund University and the Radiation protection committee at Skåne University Hospital. All experiments were performed in accordance with relevant guidelines and regulations.

### PET and MR Imaging

T1-weighted magnetization-prepared rapid gradient echo (MPRAGE) and Fluid-attenuated inversion recovery (FLAIR) images were acquired for all patients on a 3T Siemens Skyra scanner (Siemens Medical Solutions, Erlangen, Germany) and processed along with the PET images using an in-house developed pipeline, described previously^31^. Based on the FreeSurfer 5.3 (https://surfer.nmr.mgh.harvard.edu) regions derived using the Desikan-Killiany atlas larger bilateral composite ROIs (frontal and inferior frontal; medial and lateral parietal; medial and lateral temporal; and occipital cortex) were created, and to assess lateralization in patients with svPPA, a left and a right ROI was created for each lateral temporal lobe. The exact composition of these ROIs is described in the Supplementary Methods.

The radiosynthesis procedure, radiochemical purity, and scanning methods for ^18^F- Flortaucipir have been described in detail previously^32^. Subjects in this study underwent a simplified protocol including an ^18^F-Flortaucipir PET scan 80–100 min (4 × 300 s frames) post injection on a GE Discovery 690 PET scanner (General Electric Medical Systems, Milwaukee, WI, USA). PET data was processed using our in-house developed pipeline^31^. In brief, the PET data was motion corrected, summed and co-registered to the MRI data. Using the FreeSurfer segmentation of the MRI, standardized uptake value ratio (SUVR) calculations were performed using the inferior cerebellar grey matter as reference region^33^. For voxel-based analyses SUVR PET images were warped into Montreal Neurological Institute 152 standard space, the images were smoothed with an 8 mm full-width at half maximum (FWHM) Gaussian kernel. Calculations were performed using SPM12 (Wellcome Department of Cognitive Neurology, London, UK; http://www.fil.ion.ucl.ac.uk/spm) in MATLAB_R 2017b. Mean SUVR-images for AD patients and controls were calculated from non-smoothed SUVR-images in MNI-space using SPM12 and images prepared using Pmod 3.711 (Pmod Llc. Zurich, Switzerland). Individual MRI and SUVR PET-images of svPPA and *C9orf72* patients were fused and prepared using the Fusion tool in Pmod 3.711. Partial volume error correction using the Geometrical Transfer Matrix method^34^ was applied to the main data, results are presented in Supplementary Fig. 2.

### Autoradiography and immunohistochemistry

Fresh frozen blocks from temporal and frontal cortex from two patients with TDP-43 related semantic dementia and two patients with bvFTD due to expansions in the *C9orf72*-gene were kindly provided by the Dutch Brain Bank. Semantic dementia patients had TDP-43 pathology, type C, and in the *C9orf72* mutation carriers TDP-43 pathology of type B. The cases selected for autoradiography showed no or minimal tau pathology using immunohistochemistry. Ten µm sections were cut on a Leica CM3050 cryostat at −17 °C chamber temperature and −15 °C object temperature. Sections were mounted onto Histobond + microscope slides (Marienfeld Laboratory Glassware) and dried for 3 hours at room temperature before storage at −20 °C.

^3^H-Flortaucipir was tritiated at Roche with a specific activity of 33 Ci/mmol and a radiochemical purity higher than 99%. The brain sections were incubated in assay buffer (50 mM Tris buffer, pH 7.4), containing 10 nM radioligand at room temperature for 30 min. After three ten min washes at 4 °C in assay buffer and 3 quick dips in H_2_O dist. at 4 °C, the sections were dried at 4 °C for 3 h. The sections were then exposed to a FujiFilm Imaging Plate (BAS-IP TR 2025), placed in a FujiFilm Cassette (BAS 2025), for 5 days and subsequently scanned with a FujiFilm IP reader (BAS-5000) with a resolution of 25 µm per pixel. The autoradiograms were visualized with the software MCID analysis (version 7.0, Imaging Research Inc.). Non-specific binding of ^3^H-Flortaucipir was assessed by co-incubation with 10 µM unlabelled T-808. The experimental protocol was previously set up and optimized to give a robust signal in tissue sections from positive controls.

Immunohistochemistry for TDP-43 was performed on the same sections after fixation for 3 min in 100% acetone at −20 °C, and subsequent blocking for 20 min in 1% bovine serum albumin (BSA), 1% ovalbumin and 1% normal goat serum (NGS; Sigma) at RT. After rinsing in phosphate buffered saline (PBS) pH 7.4 the sections were incubated over night at 4 °C with a mouse TDP-43 antibody (CosmoBio CAC-TIP-PTD-M01, Target: TDP-43 Ser409/410; 1:1000) in PBS with 1% BSA, rinsed in PBS and incubated in an anti-mouse Alexa Fluor-488 secondary antibody (Invitrogen, A11001, 10 µg/ml) at RT for 1 hour. Sections were then stained with 4, 6-diamidino-2-phenylindole (DAPI; Roche Diagnostics) nuclear stain, rinsed in PBS and mounted prior to scanning in a Pannoramic p250 slide scanner (3DHISTECH Ltd.).

### Statistics

Statistical analyses for ROI-based comparisons were performed using GraphPad Prism 7.0a for Macintosh. For comparisons between multiple groups Kruskal-Wallis tests were used, followed by between group comparisons using Mann-Whitney tests where appropriate. Statistical significance was assumed at p < 0.05. For voxel-wise analyses of ^18^F-Flortaucipir uptake patterns between respective patient groups and control subjects we employed a voxel-wise two-sample t-test as implemented in SPM12 (http://www.fil.ion.ucl.ac.uk/spm). The voxel-wise comparisons were thresholded using family-wise error (FWE) correction with a p-value of < 0.05.

## Results

### Study participants

Six bvFTD *C9orf72* expansion carriers, six svPPA patients (three svPPA, three R-SD) and fifty-four age-matched, neurologically healthy, controls were recruited to the study.

Clinical details of the participants are given in Table 1. Structural MRI in svPPA shows various degree of atrophy in the anterior and lateral temporal lobes supporting the clinical diagnosis (Fig. 1). SvPPA cases all suffered from clinically established disease, with median symptom duration of 6 years and a CDR of 0,5 or 1. All *C9orf72* cases presented with the clinical syndrome of bvFTD, with psychotic symptoms present in 4 of the cases, and none with concomitant motor neuron disease. Transversal and coronal T1-weighted MRI images and ^18^F-Flortaucipir images for all participating svPPA and *C9orf72* patients are shown in Fig. 1.

### ROI-based analyses

**Figure 2:**
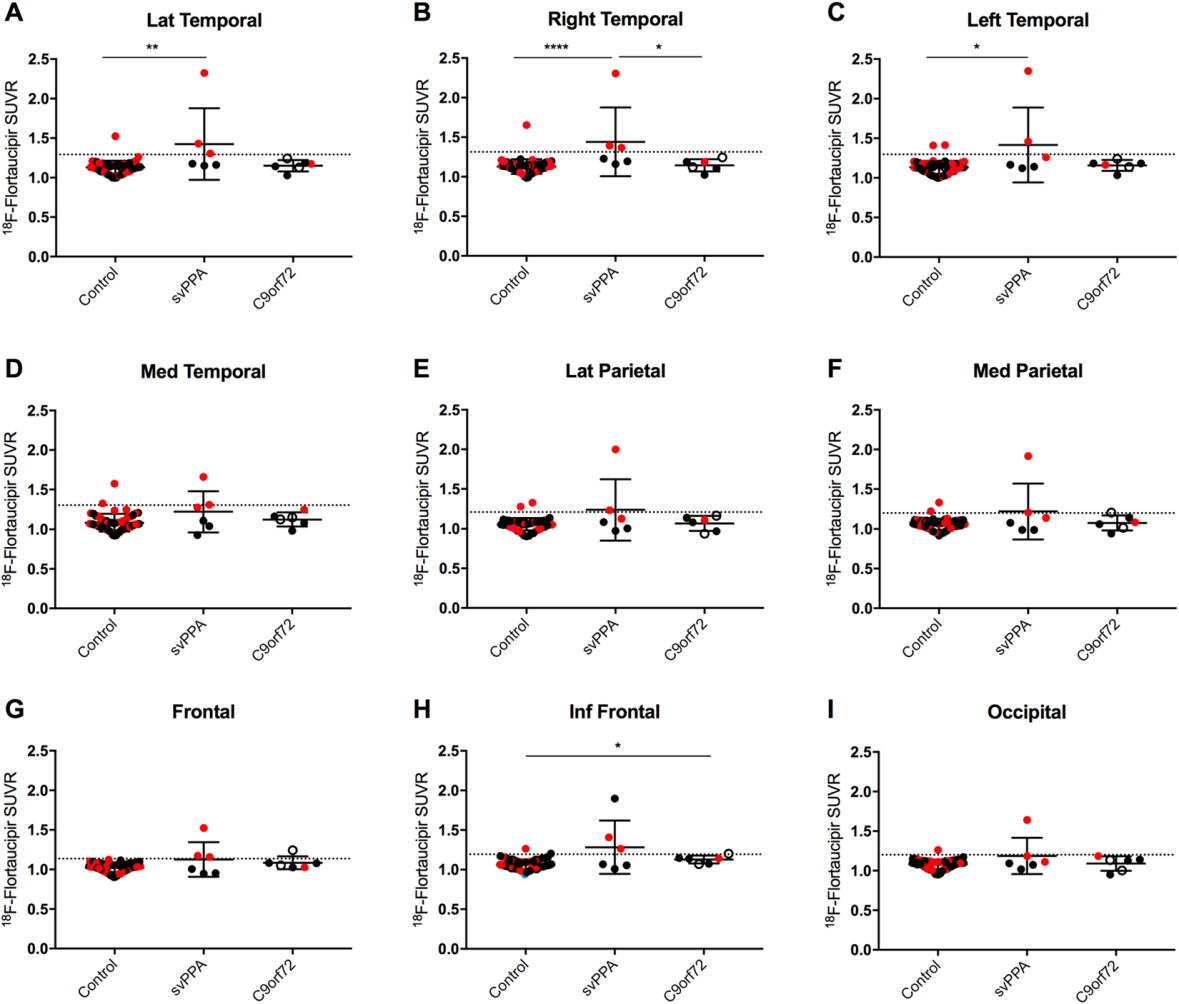
ROI-based analysis of ^18^F-Flortaucipir retention. ^18^F-Flortaucipir retention was assessed in composite ROIs consisting of the (**A**) Lateral temporal cortex (bilaterally), (**B**) Right lateral temporal cortex, (**C**) Left lateral temporal cortex, (**D**) Medial temporal cortex, (**E**) Lateral parietal cortex, (**F**) Medial parietal cortex, (**G**) Frontal cortex, (**H**) Inferior frontal cortex; and, (**I**) Occipital cortex. *p < 0.05; **p < 0.01; ****p < 0.0001. Red dots correspond to Aβ-positive individuals according to ^18^F-Flutemetamol PET scans, black dots to Aβ-negative individuals and empty circles to individuals with unknown Aβ status. Dotted lines indicates average value + 2 SD for controls.

Using ROI-based analysis significant differences were found in the lateral temporal retention of ^18^F-Flortaucipir between controls and patients with svPPA (control median 1.13 (range 0.99–1.52); svPPA median 1.24 (range 1.15–2.32), p < 0.01; Fig. 2A). In a similar fashion the lateralized temporal retention in the right and left hemispheres was significantly different comparing svPPA patients to controls (Right: control median 1.13 (range 0.99–1.65); svPPA median 1.29 (range 1.16–2.31), p < 0.0001 and Left: control median 1.13 (range 1.00–1.41); svPPA median 1.21 (range 1.12–2.35), p < 0.05; Fig. 2B,C). The retention of ^18^F-Flortaucipir in the lateral temporal lobe was higher in the more affected hemisphere, but the magnitude of increase was only in the range of a few percent (range 0–9%; Supplementary Fig. 1).

For subjects with *C9orf72*-mutations the only statistically significant difference was a slight increase of the retention in the inferior frontal lobes. The magnitude of the increase in the inferior frontal lobes was relatively small (Controls: 1.06 (range 0.94–1.26) vs C9orf72: 1.14 (range 1.07–1.20), p = 0.02; Fig. 2H). There were no significant differences in other brain regions. Since it has been shown that patients with *C9orf72*-mutations also have cerebellar dipeptide repeat and p62-pathology^35,36^, and since this pathology may interfere with the results using an inferior cerebellar reference region, we performed the same analysis using a brainstem reference. When using a brainstem reference no differences were seen in the inferior frontal lobes, but the remaining results in the *C9orf72*-mutation group were not changed (data not shown). Using partial volume error corrected data the numerical differences between groups increased and we found statistically significant differences between controls and patients carrying the C9orf72 mutation (Supplementary Fig. 2). However, except for the inferior frontal ROI all or all-but-one of the C9orf72 values were within the control mean + two standard deviations (SD) values (dotted lines), indicating that the increases in absolute values were very small.

**Figure 3:**
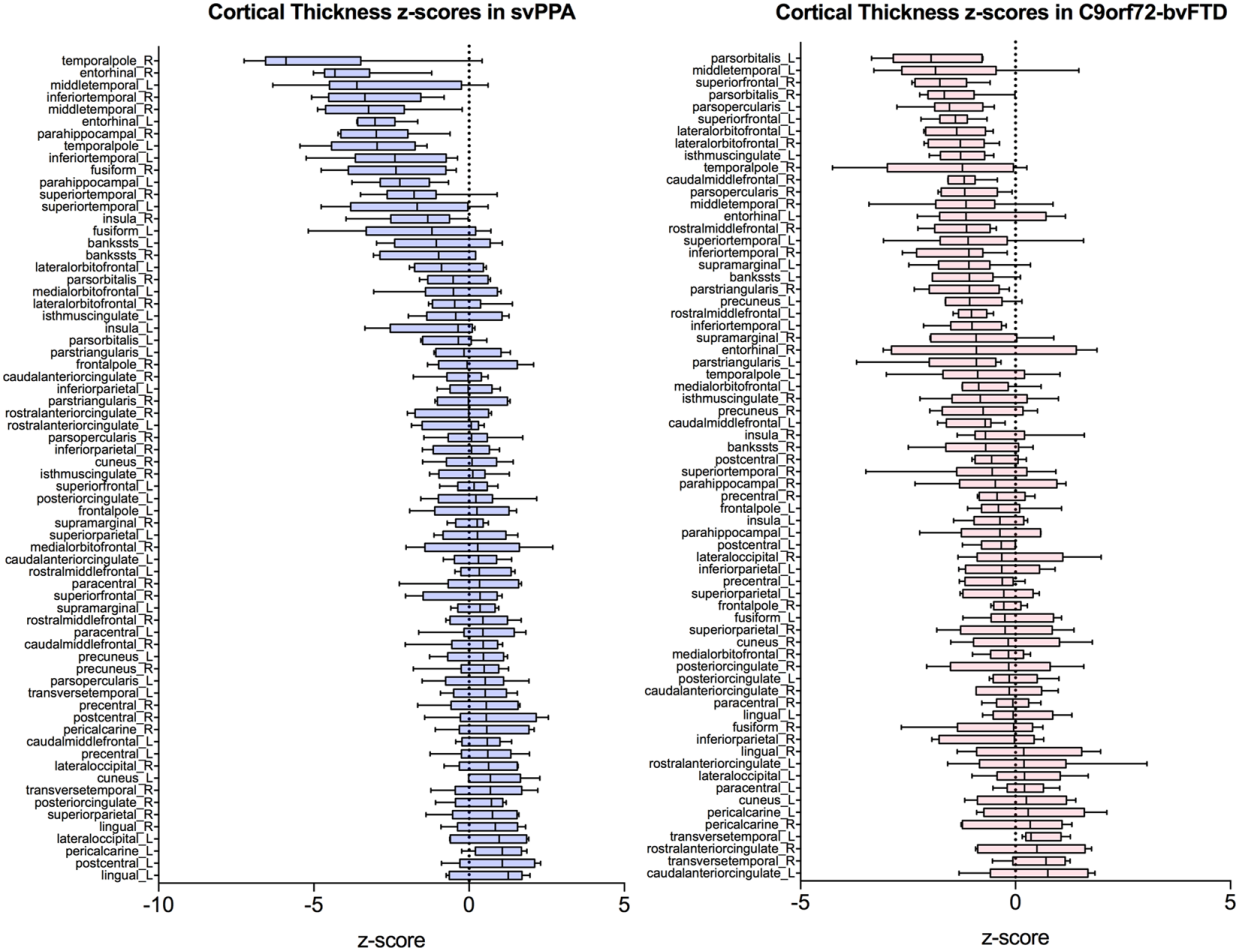
Cortical thickness z-scores for all cortical FreeSurfer regions in subjects with (**A**) svPPA, and (**B**) C9orf72-associated bvFTD. Population cortical thickness mean and standard deviations were determined in the 54 neurologically healthy normal controls. Whiskers represent 2.5–97.5 percentiles.

To compare the regional patterns of atrophy between the two disease groups the cortical thickness was calculated in each ROI. These values were normalized to z-scores using the control mean and SD and are presented in Fig. 3. We find the most severe atrophy in the anterior temporal lobes of subjects with svPPA, whereas *C9orf72*-mutation carriers instead show a more modest fronto-temporal atrophy.

### Voxel-based analyses

**Figure 4:**
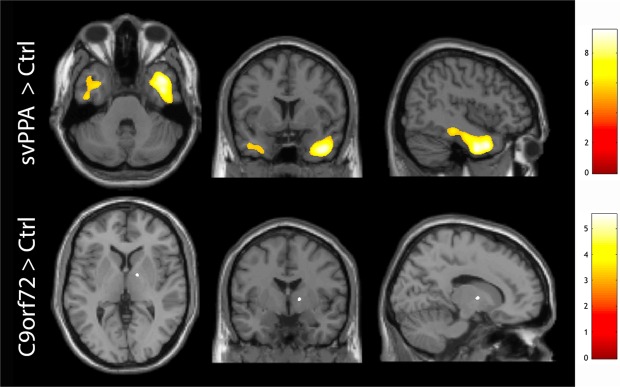
Voxel-based analysis of ^18^F-Flortaucipir retention. The upper panel shows significant voxels in the svPPA > Control contrast (p < 0.05) after correcting for family-wise errors (FWE). The lower panel shows *C9orf72* > Control contrast at p < 0.05 FWE-corrected. Scales represent t-values. Please note that the orientation of the images follows neurological convention (left in the image represents patient left).

We further performed voxel-based analyses in the svPPA and *C9orf72* groups compared to controls. In the svPPA > control contrast we could identify a large significant cluster (k_E_ = 18886; p_FWE-corr_ < 0.001) in the anterior right temporal lobe and a smaller cluster in the left temporal pole (k_E_ = 2693; p_FWE-corr_ < 0.001) after applying family-wise error (FWE) correction (Fig. 4, upper panel). In the *C9orf72*-mutation carriers > control contrast there was one small cluster located in the right posterior limb of the internal capsule (k_E_ = 45; p_FWE-corr_ = 0.03; Fig. 4, lower panel). No suprathreshold clusters were identified using the Control > svPPA or the Control > *C9orf72* contrasts. Similar results were obtained if the svPPA patient with the highest SUVR values (case 1) was excluded from the analysis (data not shown).

### Autoradiography

**Figure 5:**
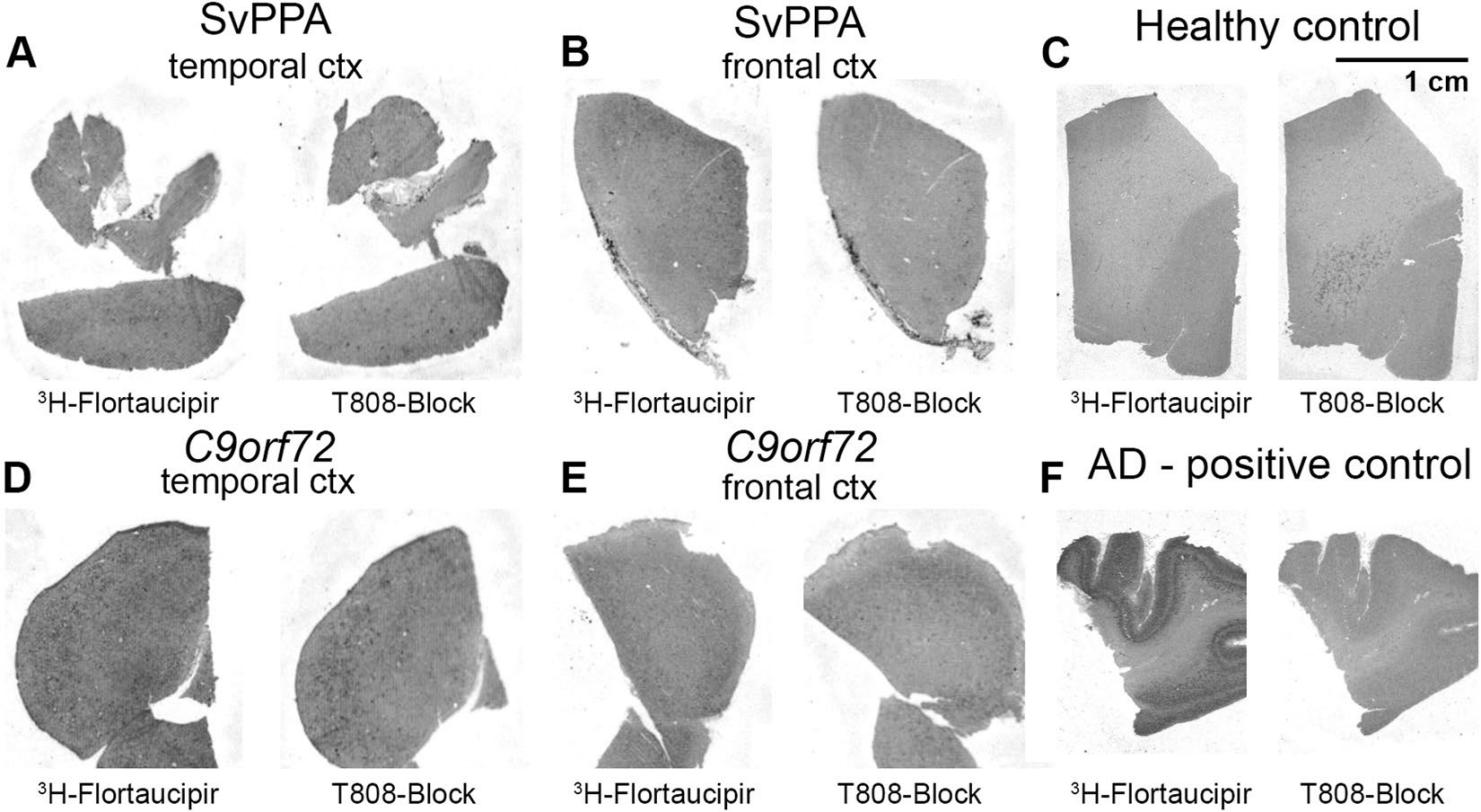
^3^H-Flortaucipir autoradiography in svPPA and *C9orf72*. Panels (A,B) show representative phosphoimaging results of ^3^H-Flortaucipir in the (**A**) temporal and (**B**) frontal cortex from a patient with svPPA and TDP-43 type C pathology. Image in (**C**) represents a negative healthy control tissue from temporal cortex. Panels (D,E) show temporal and frontal cortex respectively from a patient with *C9orf7*2-mutation and TDP-43 type B pathology. Images in (**F**) shows a positive control from AD cortex (superior temporal gyrus). For all image panels the left images show binding with ^3^H-Flortaucipir and right images remaining binding after addition of 10 µM non-radiolabeled T808. Scale indicates 1 cm.

Fresh frozen tissue samples from frontal and temporal cortex from two patients with svPPA and two with *C9orf72*-mutations, with type C and type B TDP-43 pathology respectively, were analysed using ^3^H-Flortaucipir autoradiography. No specific binding of ^3^H-Flortaucipir to TDP-43 pathology could be detected in the cortical tissue sections in these tissue samples (Fig. 5A–E). A grainy autoradiography pattern was sometimes seen (Fig. 5D), but this signal could not be blocked by high concentration unlabeled T-808 and the grains did not colocalize with TDP-43 positive structures using TDP-43 immunohistochemistry (Supplementary Fig. 3). In a positive control from AD cortex (superior temporal gyrus) a clear positive ^3^H-Flortaucipir signal, that was blocked after addition of cold T-808 compound, was seen in several cortical layers (Fig. 5F).

## Discussion

We present data showing increased retention of ^18^F-Flortaucipir in the lateral temporal lobes of left-sided svPPA and in the homologous right-sided SD using a ROI-based analysis. These results are in line with previously reported findings of increased binding potential (BP_ND_) of ^18^F-Flortaucipir in patients with svPPA/R-SD^19,20^, and consistent with the expected distribution of pathology in svPPA. At a voxel-wise level, significantly increased clusters in svPPA were located in the anterior temporal poles bilaterally, confirming the location of the ROI-based data. The underlying molecular pathology of svPPA/SD is most often type C TPD-43 pathology, but the syndrome can also be caused by AD pathology or 3R tau pathology (Pick’s Disease)^1,3,37^. The Aβ status among the svPPA patients shows that three of the patients in this study have pathological levels of Aβ (Table 1). The β-amyloid-PET values are, however, borderline positive despite the patients having had disease symptoms for many years. We believe that the patients should have shown more aberrant values of Aβ, if the patients would have had their symptoms primarily due to AD pathology. Other notions that would support other causes than AD being the underlying pathology in these cases is that, apart from case 1, the magnitude of increase in ^18^F-Flortaucipir SUVR is low in comparison to the retention seen in symptomatic AD (see Supplementary Fig. 4 for comparison); and, patterns of cortical atrophy showing a relatively isolated anterior temporal lobe atrophy in svPPA-cases. Further, the previously published findings of increased anterior lateral temporal ^18^F-Flortaucipir retention in four^19^ and five^20^ Aβ-negative subjects from two independent research groups support the idea that there is an Aβ-independent increase in ^18^F-Flortaucipir retention in svPPA-cases. Another theoretically possible explanation is the phenomenon of mixed TDP-43 and tau pathology, or concomitant “secondary” tau pathology, however this appears to be a very rare phenomenon^37^.

The finding of increased ^18^F-Flortaucipir retention in the svPPA disease group has led to the questioning of the selectivity of the ^18^F-Flortaucipir tracer for tau pathology, and off-target binding to TDP-43 has been suggested^19^. We therefore recruited six patients with bvFTD due to hexanucleotide expansions in the *C9orf72* gene. The *C9orf72* gene mutation is strongly associated to TDP-43 pathology of subtypes A and B^4,38^ and the likelihood of these patients having TDP-43 pathology is thus high. In the ROI-based analysis of *C9orf72* mutation carriers we did find a significantly increased retention of ^18^F-Flortaucipir in the inferior frontal cortex, that could potentially fit with a behavioural variant FTD, but the magnitude of ^18^F-Flortaucipir retention increase was low. This effect was not visible on voxel-based analysis and disappeared when using the pons as an alternative reference region. In the voxel-wise analysis of *C9orf72*-mutation carriers the only area that was statistically higher than in controls was a small cluster in the posterior limb of the internal capsule, the significance of this cluster is uncertain, but it colocalizes to a region with atrophy on MRI-based measures in *C9orf72* patients^36^. Apart from these regions no regions or voxels with increased ^18^F-Flortaucipir retention were detected, including the cerebral cortex and hippocampus, where TDP-43 pathology is prominent in *C9orf72* carriers^35,36,38^. A previous case report has shown an increased temporal retention of Flortaucipir in a subject with a *C9orf72* mutation and a three year history of personality change^21^. The disease duration of the published subject is similar to the disease duration of the participants in this study. We could not reproduce this finding in our six patients. The Aβ-status of the published subject is unfortunately unknown and a concomitant AD can not be excluded.

Previous autoradiography studies have shown no^13^ or minimal^12,15^ binding of ^18^F-Flortaucipir to *post mortem* tissue from TDP-43 related FTD cases. In these studies only cases with TDP-43 pathology of types A and C were included. In our autoradiography results we show no specific binding of ^3^H-Flortaucipir to cortical tissue from *C9orf72*-mutation carriers having a type B TDP-43-pathology. In a similar fashion we found no specific binding to TDP-43 type C pathology in tissue from svPPA cases.

Taken together, our results do not support the view that the increased retention of ^18^F-Flortaucipir seen in svPPA would be due to a general off-target binding to TDP-43 pathology. It is possible that ^18^F-Flortaucipir might have an off-target binding to TDP-43 type C pathology, but the finding of no, or only minimal, binding of ^3^H-Flortaucipir to type C TDP-43 pathology in autoradiography experiments would argue against that notion. The possibility of ^18^F-Flortaucipir binding to monoamine oxidase B (MAO-B), similar to the MAO-B-binding of THK5351^39^ has been suggested^19,20^, but recent studies have shown that there is no reduction of ^18^F-Flortaucipir retention in patients taking MAO-B inhibitors^40,41^. Since the regions where the most intense ^18^F-Flortaucipir retention is seen are regions with a pronounced atrophy a possible cause for the increased retention could be an off-target binding to another neurodegenerative process that parallels the atrophy in svPPA. Possibly, the severe atrophy seen in the temporal poles in svPPA may damage the blood-brain barrier and cause an increased retention of the ^18^F-Flortaucipir tracer. Since the tracer kinetics of Flortaucipir differs between off-target binding regions, such as the putamen, and binding to AD tau pathology in the cerebral cortex^32^ future kinetic studies may shed light on the nature of the cortical tracer retention in svPPA. Apart from the retention in the temporal lobes of svPPA-cases no other regions of unexpected off-target binding were seen in svPPA or *C9orf72*-mutation carriers.

A limitation of the present study is the low number of participants in the respective disease groups, and the results should be interpreted with this in mind. Another limitation is the absence of neuropathological confirmation of the diagnoses and direct neuropathological correlations of pathology to PET-retention. In svPPA the majority of cases are related to type C TDP-43 pathology, but despite determination of Aβ-status using CSF or PET other underlying pathologies, such as AD and 3R tau, cannot fully be excluded in our study. Further, the C9orf72-cases have a shorter disease duration and a lower degree of atrophy compared to the svPPA cases. We cannot exclude the possibility that there could be an increased retention in more advanced disease also in C9orf72 mutation carriers.

In conclusion, we find an increased ^18^F-Flortaucipir retention *in vivo* in the lateral temporal cortices of patients with svPPA/R-SD. In patients with mutations in the *C9orf72*-gene, only very limited ^18^F-Flortaucipir retention could be detected, indicating that binding of ^18^F-Flortaucipir in TDP-43 proteinopathies is not a general TDP-43 related phenomenon.

## Supplementary information

Supplementary information
