# Supplementary material for: 18F-Flortaucipir in TDP-43 associated frontotemporal dementia
Source: Sci Rep. 2019 Apr 15;9:6082. doi: 10.1038/s41598-019-42625-9 (PMC6465310; doi:10.1038/s41598-019-42625-9)
Supplement: Supplementary file 1 — Supplementary information [file 41598_2019_42625_MOESM1_ESM.pdf]

Supplementary information

^{18}F-Flortaucipir in TDP-43 associated frontotemporal dementia

Smith R, Santillo A F, Landqvist Waldö M, Strandberg O, Berron, D, Vestberg S, van Westen D, van Swieten J, Honer M, and Hansson O.

Supplementary methods:

Regions of interest
Composite regions of interest (ROIs) were created based on FreeSurfer 5.3 segmentation of the MRI using the Desikan-Killiany atlas as indicated below, regions were pooled weighted according to their MR voxel volume.

Lateral parietal: Bilateral inferiorparietal, superiorparietal and supramarginal ROIs.

Medial Parietal: Bilateral isthmuscingulate and precuneus ROIs.

Lateral Temporal: Bilateral bankssts, inferiortemporal, middletemporal, superiortemporal, transversetemporal ROIs.

Medial temporal: Bilateral entorhinal and parahippocampal ROIs.

Frontal: Bilateral caudalmiddlefrontal, parsopercularis, parsorbitalis, parstriangularis, rostralmiddlefrontal, superiorfrontal, and frontalpole ROIs.

Inferior Frontal: Bilateral medialorbitofrontal and lateralorbitofrontal ROIs.

Occipital: Bilateral cuneus, lateraloccipital, lingual and pericalcarine ROIs.

Left Temporal: Left bankssts, inferiortemporal, middletemporal, superiortemporal and transversetemporal ROIs.

Right Temporal: Right bankssts, inferiortemporal, middletemporal, superiortemporal and transversetemporal ROIs.

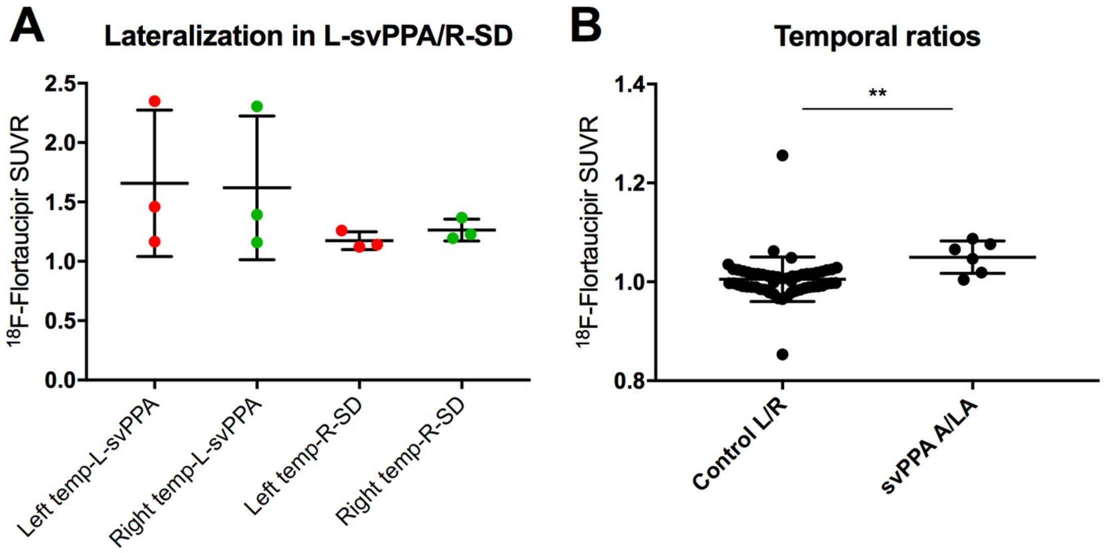

Supplementary Figure 1. Panel A shows the individual retention in the left and right temporal lobes of left-sided svPPA (L-svPPA) and right dominant semantic dementia (R-SD). Panel B shows the ratio between left and right sides in controls (L/R) and the affected (A) over less affected (LA) side in L-svPPA and R-SD cases. ** indicates $p < 0.01$.

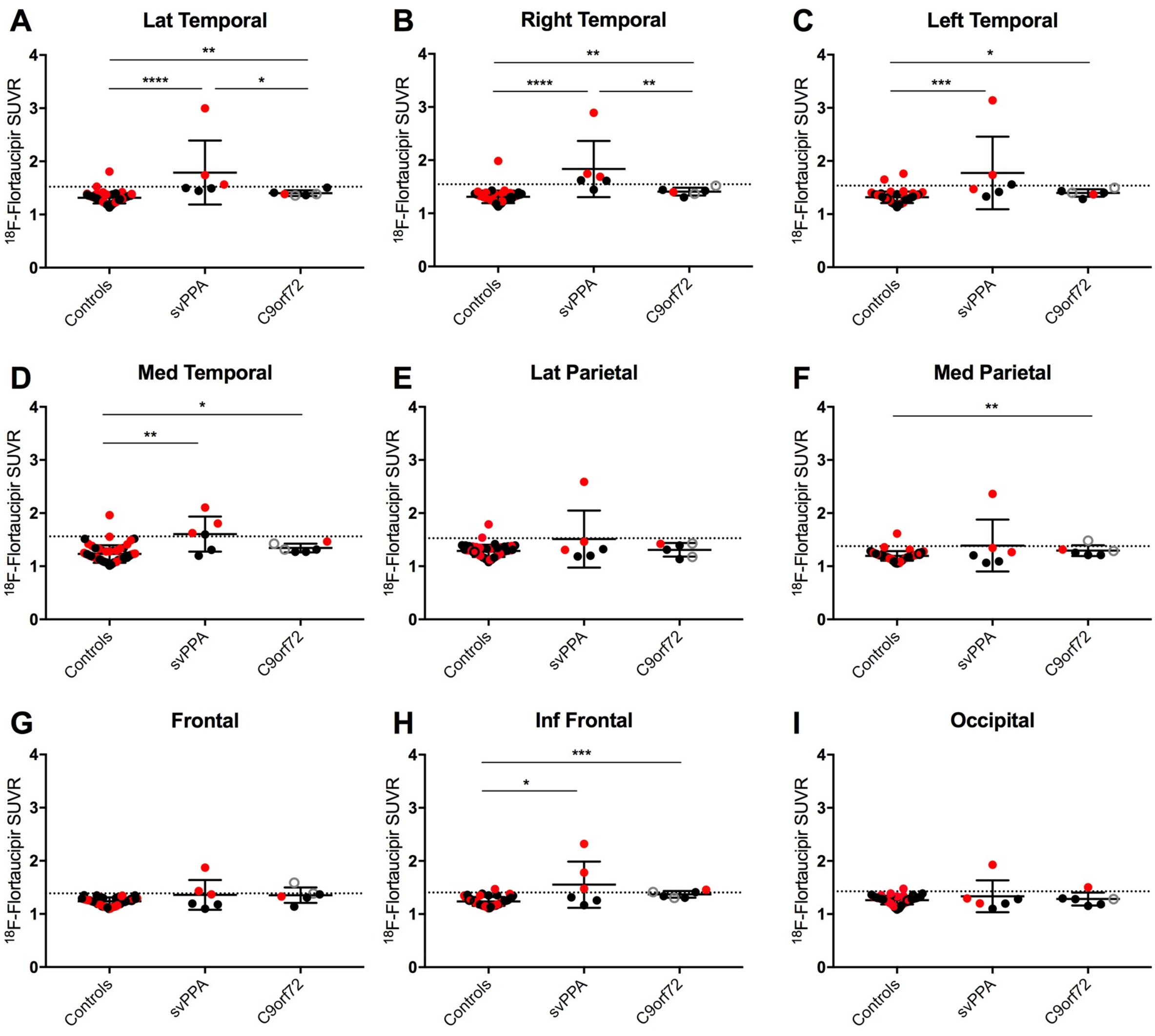

Supplementary Figure 2. Partial Volume Error corrected data using the Geometric Transfer Matrix method. ROIs similar to Figure 2 were used. * indicates $p < 0.05$, ** indicates $p < 0.01$, *** indicates $p < 0.001$, **** indicates $p < 0.0001$. Dotted lines indicates the control means + 2 standard deviations. Red dots indicate β-amyloid positive subjects, Black dots β-amyloid negative subjects. Circles subjects with unknown β-amyloid status.

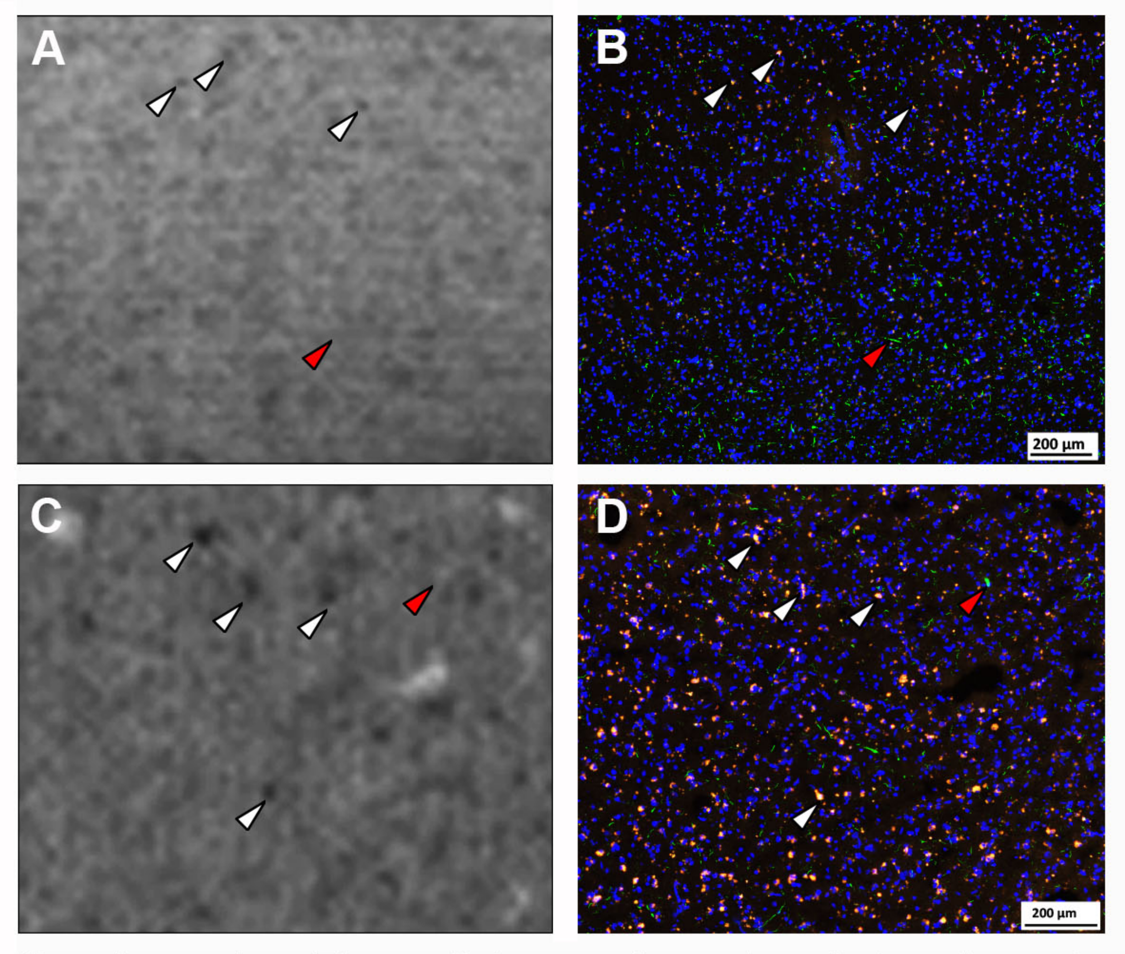

Supplementary Figure 2. Images from phosphoimaging auto-radiography (ARG; A, C) and immunohistochemistry (IHC; B, D) in the same sections from a case with svPPA (A, B) and a subject carrying a C9orf72 mutation (C, D). In IHC TDP-43 is colored green, lipofuscine (orange) and cell nuclei in blue. White arrowheads show lipofuscine-positive inclusions in IHC with corresponding locations in ARG images. Red arrowheads show TDP-43 positive inclusions. Scale bars indicate 200 µm.

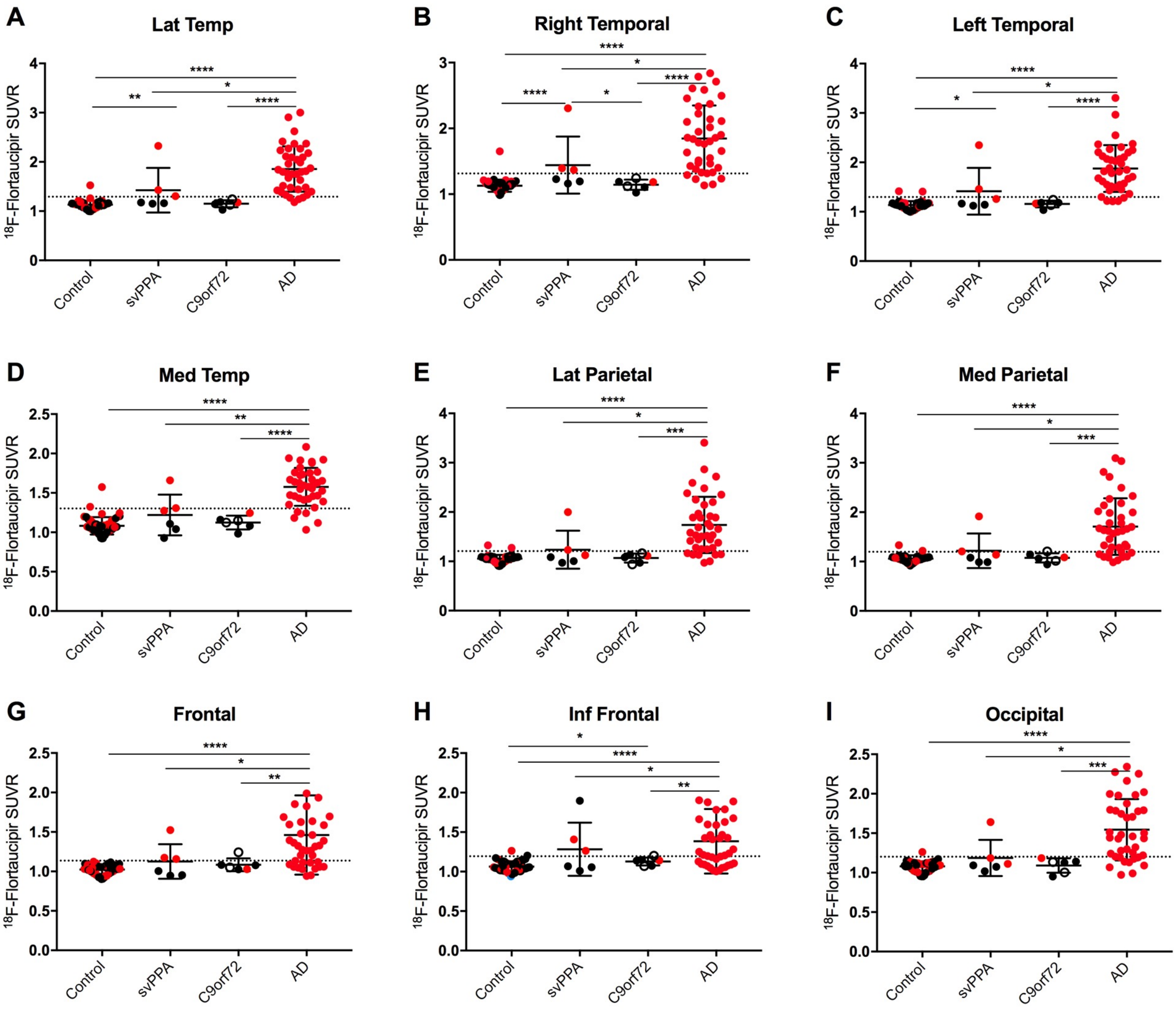

Supplementary Figure 4. ROIs similar to Figure 2 were used. Values derived from 39 AD patients are included for comparative purposes. * indicates $p < 0.05$, ** indicates $p < 0.01$, *** indicates $p < 0.001$, **** indicates $p < 0.0001$. Dotted lines indicates the control means + 2 standard deviations. Red dots indicate β-amyloid positive subjects, Black dots β-amyloid negative subjects. Circles subjects with unknown β-amyloid status.

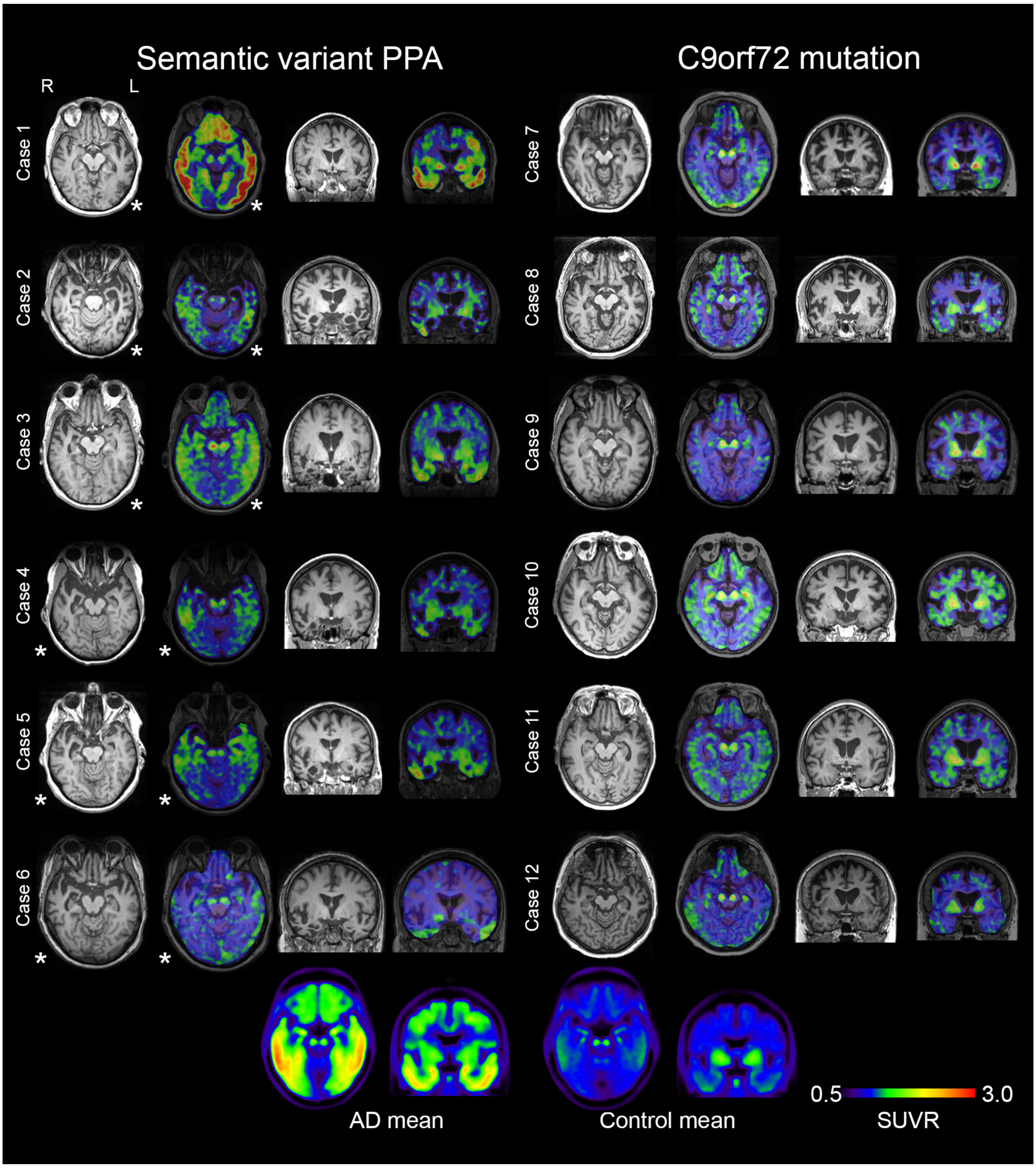

Semantic variant PPA
C9orf72 mutation
R
L
Case 1
Case 2
Case 3
Case 4
Case 5
Case 6
Case 7
Case 8
Case 9
Case 10
Case 11
Case 12
AD mean
Control mean
0.5
3.0
SUVR
